# Smoking patterns and the intention to quit in German cancer patients: a cross-sectional study

**DOI:** 10.1186/s12885-024-12380-w

**Published:** 2024-06-06

**Authors:** Frederike Bokemeyer, Lisa Lebherz, Carsten Bokemeyer, Kathleen Gali, Holger Schulz, Christiane Bleich

**Affiliations:** 1 Department of Medical Psychology, University Medical Center Hamburg Eppendorf, Martinistraße 52, 20246 Hamburg, Germany; 2 Center for Oncology, II. Medical Clinic and Polyclinic, University Medical Center Hamburg Eppendorf, Martinistraße 52, 20246 Hamburg, Germany; 3 Cancer Epidemiology Group, University Cancer Center Hamburg (UCCH), University Medical Center Hamburg-Eppendorf (UKE), Martinistraße 52, 20246 Hamburg, Germany; 4 Hamburg Center for Health Economics (HCHE), University of Hamburg, Esplanade 36, 20354 Hamburg, Germany

**Keywords:** Cancer, Smoking cessation, Psycho-oncology

## Abstract

**Background:**

Continued smoking after a cancer diagnosis can be associated with lower treatment tolerance, poorer outcomes, and reduced quality of life compared to non-smoking cancer patients or to those who have quit. Yet about 60% of patients continue to smoke after being diagnosed and find it difficult to quit. To address this problem, it is necessary to identify current and past smoking patterns (e.g., frequency of use, types of tobacco products) and determine whether there is motivation to quit. Similarly, factors associated with continued smoking should be identified. These data will provide the basis for the development of smoking cessation programs tailored to the needs of cancer patients.

**Methods:**

A questionnaire was distributed to cancer patients older than 18 years in a German Comprehensive Cancer Center. Participating cancer patients were divided into three main groups: 1) patients who stopped smoking before being diagnosed with cancer (Ex-before); 2) patients who stopped smoking after a cancer diagnosis (Ex-after); and 3) patients who currently smoke cigarettes (CS). Sociodemographic, medical, and psychosocial data were collected, as well as smoking patterns and the motivation to quit smoking.

**Results:**

About half of patients (51%) who smoked before diagnosis continue to smoke after a cancer diagnosis. Being diagnosed with a tobacco-related cancer type was associated with a decreased probability of continued smoking. Patients with tobacco-related tumors and receiving positive support in burdensome situations were more likely to have a higher cigarette dependence. Of all CS, 59.1% had intention to quit, and 22.7% reported having taken action to quit.

The support by a smoking cessation program was considered important. CS were willing to spend up to €100 for support and were open to multiple sessions per week, group sessions, one-on-one sessions and/or online support.

**Conclusion:**

These findings underscore the importance of educating cancer patients about the consequences of smoking and to provide them with support to quit. Identified risk factors may further help to recognize cancer patients with high risk of continued smoking after diagnosis.

**Trial Registration:**

The study was registered at OSF (https://osf.io/3c9km) and published as a study protocol at “https://bmjopen.bmj.com/content/13/4/e069570”.

**Supplementary Information:**

The online version contains supplementary material available at 10.1186/s12885-024-12380-w.

## Background

Cancer is one of the leading causes of death worldwide, accounting for nearly 10 million deaths in 2020. Smoking is a risk factor for almost all types of cancer and is responsible for two-thirds of lung cancer deaths [1].

Once a person has been diagnosed with cancer, continued smoking can lead to significant negative health and treatment outcomes compared with non-smoking cancer patients. Adverse outcomes include poorer wound healing after surgery [2], reduced efficacy and poorer outcome after radiotherapy [3], or systematic therapy [4] and more side effects such as pain [5] and fatigue [6]. In addition, cancer patients who smoke have twice the risk of heart attack, stroke or death from cardiovascular disease compared to non-smokers [7] and their long-term survival may be reduced [8, 9]. Tao et al. 2013 [10] showed in a Shanghainese cohort study, that the median survival time after cancer diagnosis of patients who continued to smoke was 2.1 years, compared with 4.4 years for patients who had quit. Furthermore, continued smoking increases the likelihood to develop a secondary primary tumor [8], metastases or recurrences [9]. Finally, cancer patients who quit smoking report a better quality of life and also lower depression scores [11].

The importance of educating patients about these consequences as well as motivating and supporting them to quit smoking is clear [12]. However, up to 60% of cancer patients who have smoked before diagnosis continue to smoke [13].

The Transtheoretical Model (TTM) of behavioral change can be used to describe and assess patients’ motivation to quit smoking and has been validated in empirical studies and has demonstrated usefulness and practicality [14]. According to this model, the path from smoking to non-smoker consists of several successive stages: 1) pre-contemplation, 2) contemplation, 3) determination, 4) action, 5) maintenance. During the transition from one phase to the next, affective processes and behavioral adaptions play an important role. Only someone who has reached the last stage of maintenance can be considered not smoking. However, it is possible to return to earlier stages and go through the cycle several times.

A variety of smoking cessation interventions have been developed in recent years to help cancer patients quit smoking. Unfortunately, recent meta-analyses show that the success of interventions tailored to cancer patients is insufficient [15]. A major reason for low success rates of smoking cessation programs in oncology patients may be that the specific and complex needs of cancer patients compared to the general population of people who smoke are not adequately addressed.

Factors that have been shown to be associated with smoking patterns in cancer patients include several different factors such as age [16], level of education [17]; type of diagnosed tumor [16]; alcohol consumption [18], and received social support [19]. In the population of non-cancer smokers, even more associated factors were found, such as relationship [20] and having children [21]. These factors have not yet been tested for their association with different smoking patterns in cancer patients. All of these factors will be analyzed in this study. (For more detailed information on all of the factors mentioned, see the study protocol at “https://bmjopen.bmj.com/content/13/4/e069570”) In addition, to our knowledge, this analysis is the first to examine the relationship between cancer patients' smoking patterns and existing knowledge about the consequences of continued smoking after cancer. The present study is intended to provide an exploratory basis for the development of a smoking cessation program tailored to the specific situation of cancer patients.

### Research Questions (RQ)

The following research questions were analyzed as part of the study:

What is the proportion of cancer patients who smoke, and how can their smoking patterns be characterized (level of cigarette dependence, level of motivation to quit, products smoked, smoking breaks, amount smoked per day, and total years smoked)?

What sociodemographic, medical, and psychological factors are associated with current smoking status after a cancer diagnosis?

What sociodemographic, medical, and psychological factors are associated with the level of cigarette dependence in current smoking cancer patients?

What is the proportion of cancer patients who continue to smoke in each motivational stage of the adapted version of the TTM (lack of intention, intention formation and action), and what sociodemographic, medical, and psychological factors are associated with each stage?

What is the perceived need for a specific smoking cessation program for cancer patients and how should this program be designed?

## Methods

### Design

This multicenter cross-sectional study examined smoking patterns, smoking cessation motivation, and risk factors for smoking continuation after cancer diagnosis among cancer patients undergoing diagnosis, treatment, or follow-up in the catchment area of a Cancer Center in a German metropolitan region. The results are based on a written survey of cancer patients over 18 years of age. More details can be found in the study protocol (https://bmjopen.bmj.com/content/13/4/e069570) [22].

### Participants

Inclusion criteria for study participation are:

- being over 18 years of age,
- being diagnosed with any type of malignant tumor,
- having sufficient knowledge of the German language, and
- being in any stage of cancer treatment (including follow-up).

Participants were split into three main groups by a filter question in the questionnaire: Never smokers (NS), former smokers (EX-before/EX-after), and current smokers (CS), with former smokers further subdivided by timing of smoking cessation in relation to the date of their cancer diagnosis:

- Never smokers (NS): Participants who have smoked fewer than 100 cigarettes or other smoking products in their lifetime.
- Ex-smokers, who quit before cancer diagnosis (Ex-before): Participants who have smoked more than 100 cigarettes or other smoking products in their lifetime but quit before the cancer diagnosis and are currently not smoking.
- Ex-smokers, who quit after cancer diagnosis (Ex-after): Participants who have smoked more than 100 cigarettes or other smoking products in their lifetime but quit after the cancer diagnosis and are currently not smoking.
- Current smokers (CS): Participants who have smoked more than 100 cigarettes or other smoking products in their lifetime and are current smokers.

Power calculations based on RQ1 indicate that a sample size of at least *N*=865 would yield a two-sided 95%confidence interval with a width of 4%, assuming that the proportion of current smokers in the sample is approximately 10%.

### Recruitment and procedure

Recruitment of cancer patients took place in various inpatient and outpatient clinics. They were approached in five clinics of the University Medical Center (oncology ward, otolaryngology ward, radiotherapy ward, gynecology outpatient clinic, oncology outpatient clinic) as well as in two cooperating private practices and hospitals. The oncology ward and outpatient Clinic offer diagnosis, treatment, and follow-up care for all types of cancer. The gynecology outpatient clinic specialized in breast tumors and female genital tract tumors, such as uterine or ovarian cancer. The otolaryngology outpatient clinic specialized in head and neck cancers. In the department of radiotherapy, the research assistants came into contact with patients with various cancer diagnoses who were receiving radiotherapy. Finally, the cooperating private practices and hospitals in our network focused on lung and prostate cancer patients. There were no incentives or any compensation for participation. The eligibility of potential participants was verified by our research assistants. Prior to participation, all participants received information about the study and completed an informed consent form. This consent form was kept separate from the completed questionnaire so that no conclusions could be drawn about each individual. This ensured anonymity and reduced social desirability bias. Reasons for declined participation of eligible patients were recorded**.** This study was approved by the Local Psychological Ethics Committee of the Center for Psychosocial Medicine Hamburg (LPEK) (tracking number: LPEK-0212).

### Measures

A paper-pencil questionnaire consisting of validated instruments and self-developed items was compiled. The questionnaire is a self-report instrument that was completed by cancer patients without structured assistance. It consisted of different parts for each target group (i.e., NS, EX-before/after and CS).

Sociodemographic data (gender, age, relationship, living situation, education level and employment status*)* as well as medical data (cancer type, recurrences, current, planned and completed treatments, and comorbidities and other medical conditions) were collected. A distinction was made between tobacco-associated and non-tobacco-associated cancers (based on the relevant literature, classification was made by two physicians). The following cancers were classified as tobacco-related: pancreas, ovarian, urinary bladder, liver, biliary tract, oral cavity/pharynx/larynx, gastric, lung, kidney, esophageal.

Two items from the EORTC QLQ C30 (European organization for research and treatment of cancer quality of life questionnaire) were used to assess self-reported health status and health-related quality of life (HRQOL) [23, 24]. To assess passive smoking, two items from the German Health Survey 1998 (BGS98) have been added [25]. To assess knowledge of the consequences of continued smoking, an 8-item questionnaire “Knowledge regarding the consequences of continuing to smoke after cancer diagnosis “(KSC-8) was developed (see Additional file 1). On a five-point Likert response scale, patients could choose between “I do not agree at all”, “I do not agree”, “I partially agree”, “I agree”, and “I completely agree”. Social support was assessed using the German SSUK-8 (Social Support - Cancer Patients) [26]. It consisted of eight items measuring positive support (4 items) and negative interactions (4 items). The 3-item “Audit-C” (Alcohol Use Disorders Identification Test-Consumption) [27] was used to measure alcohol consumption. The German version of the Distress Thermometer was used to assess distress in cancer patients [28]. Items from the German National Cohort (GNC) questionnaire were used to obtain information on current smoking patterns such as product smoked, amount smoked, and frequency of smoking [29]. The self-developed OSCC (Opinion on a smoking cessation program for cancer patients) was used to ask former and current smokers about their thoughts on a potential smoking cessation program for cancer patients (see Additional file 2). It consisted of four quantitative items for former and current smokers (e.g., the importance of education, the usefulness of a smoking cessation program for cancer patients and potential participation). The items had five response options, ranging from “not at all true” to “very true”. For current smokers, the instrument also included five items assessing logistic preferences for a smoking cessation program (e.g., preferred time, frequency, and setting). The German 6-item version of the Fagerström Test for Cigarette Dependence (FTCD) was used to assess potential cigarette dependence in current smokers [30]. It should be noted that this test has only been validated for cigarette use. Patients who smoked only alternative products were excluded from its evaluation. To measure the willingness to quit smoking, the German Intention to Quit Smoking questionnaire (FÄR) was used [31], which is based on the modified TTM [14] and assessed three motivational smoking cessation stages i.e., lack of intention, intention formation, action.

A pilot test was conducted with seven cancer patients prior to the start of recruitment. They completed the questionnaire under the supervision of a research assistant and were asked to verbalize their thoughts aloud [32].

Methodological details of the research project can be found in the published study protocol (https://bmjopen.bmj.com/content/13/4/e069570) [22].

### Statistical analysis

Descriptive statistics were computed to describe patient characteristics with respect to sociodemographic and medical variables of the subgroups. Categorical data were summarized by absolute and relative frequencies. Continuous data were summarized by means and standard deviations (SD). Different research questions were analyzed using the appropriate subsample. Descriptive statistics of items measuring *current smoking patterns* (Research question 1, RQ1) were performed to assess the proportion and smoking pattern of CS in our sample. RQ2 was answered using a multiple logistic regression, comparing CS with EX-after (binary variable). Predictors included in the model were: *Gender, age, highest level of education, relationship, having children, tobacco-associated cancer type, alcohol consumption, and social support*.

To answer RQ3 a multinomial logistic regression was conducted to predict the level of *dependence* among current smokers, using the same predictors as in RQ2.

A multinomial regression model was used to identify predictors of the three levels of the *motivation to quit smoking* (RQ4, lack of intention, intention formation, action) among CS. Predictors used in this model were: *gender, age, relationship, having children, tobacco-associated cancer type, alcohol consumption, and knowledge of the consequences of continuing smoking*. The reference category was patients scoring on “action” on the TTM. Finally, four items on the need for a smoking cessation program and five items for CS on their preferences for the design of such a program were analyzed using descriptive statistics (mean, SD) (RQ5).

All statistical analyses were performed using SPSS version 27.0 (IBM Corp). Missing data were imputed using the expectation maximization algorithm. Cases missing more than 30% of all variables were excluded from the analysis [33]. For inferential statistics, findings with p ≤.05 were considered as statistically significant. To test the robustness of the results, we performed sensitivity analyses using only complete cases (without imputation of missing values).

## Results

### Sample characteristics

**Fig. 1:**
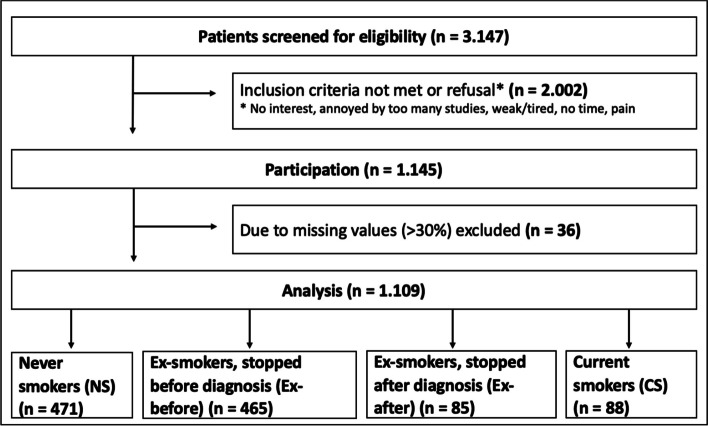
Patients screened, excluded and enrolled

From a total of 3147 screened patients, 1145 patients were enrolled in this study resulting in a participation rate of 36.4%. Reasons for refusal to participate included “not interested”,” annoyed by being asked to participate in too many studies”, ”too weak/tired”, ”no time” or ”experiencing pain“. For 36 patients the proportion of missing values exceeded 30%. A total of 1109 patients were included in the analyses (Fig. 1).

### Sociodemographic characteristics

The mean age of the sample is 61.01 (SD=11.9) and 22.1% are female. Almost half of the sample reported being former smokers.

Regarding the sociodemographic characteristics of the patients, 83.3% of the patients reported being in a current relationship and 59.1% of the patients had completed the highest level of education. Regarding the employment status of the patients, 45.4% reported to be employed and another 40.4% reported to be retired. Complete sociodemographic data for the four subgroups are shown in Table 1.

**Table 1:** Sociodemographic characteristics of the sample by subgroup

	**NS** **(*N* = 471)**		**EX-before** (***N* = 465)**		**EX-after** **(*N* = 85)**		**CS** **(*N* = 88)**	
**Age**. Mean (SD)	59.89 (13.43)		63.63 (9.54)		55.85 (12.33)		58.21 (10.84)	
	**N**	**%**	**N**	**%**	**N**	**%**	**N**	**%**
**Gender**								
Female	112	23.8	82	17.6	34	40.0	17	19.3
**Education**								
Four to nine years of school	51	10.8	75	16.1	12	14.1	20	22.7
Ten years of school	116	24.6	115	24.7	35	41.2	24	27.3
High school diploma: 12–13 years of school	302	64.1	271	58.3	38	44.7	44	50.0
**Relationship**								
In a relationship	395	83.9	397	85.4	62	72.9	70	79.5
**Employment**								
Unemployed	10	2.1	9	1.9	6	7.1	3	3.4
Employed	158	33.5	103	22.2	36	42.4	31	35.2
Self employed	73	15.5	76	16.3	8	9.4	18	20.5
Retired	164	34.8	230	49.5	28	32.9	26	29.5
Other	65	13.7	47	10.1	7	8.2	10	11.4
**Living situation**								
Alone	74	15.7	69	14.8	19	22.4	20	22.7
With partner	262	55.6	324	69.7	45	52.9	45	51.1
With partner and children	105	22.3	60	12.9	14	16.5	19	21.6
Other	30	6.4	12	2.6	7	8.2	4	4.5

Due to missing data, does not always sum to total sample

### Clinical characteristics

The data show that 69.0% of patients surveyed were currently receiving treatment, while 37.1% of patients had already completed their treatment and 20.4% were scheduled for treatment. Note that these treatment phases are not mutually exclusive. The majority of patients were diagnosed with cancer of the urogenital tract (58.5 %) and a very limited number of patients were diagnosed with head and neck cancer (1.4%). Regarding comorbidities, 56.8% of patients reported having at least one other disease besides cancer. Of all patients, 24.7% reported being regularly exposed to secondhand smoke in at least one relevant place (home, at work). See Table 2 for descriptive medical data for the four groups.

**Table 2:** Clinical characteristics by subgroup

	**NS** **(*N* = 471)**		**EX-before** **(*N* = 465)**		**EX-after** **(*N* = 85)**		**CS** **(*N* = 88)**	
	N	%	N	%	N	%	N	%
**Type of Cancer**								
Gastrointestinal	9	1.9	10	2.2	5	5.9	3	3.4
Breast	22	4.7	16	3.4	5	5.9	2	2.3
Urogenital	283	60.1	295	63.4	20	23.5	51	58.0
Gynecological	19	4.0	11	2.4	4	4.7	3	3.4
Blood cancer	12	2.5	7	1.5	1	1.2	3	3.4
Head and neck tumors	5	1.1	5	1.1	5	5.9	1	1.1
Lung cancer	20	4.2	45	9.7	19	22.4	9	10.2
Lymphomia	27	5.7	25	5.4	5	5.9	2	2.3
Unknown	1	0.2	2	0.4	0	0	1	1.1
Other	73	15.5	49	10.5	21	24.8	13	14.8
**Treatment**^bc^								
Currently being treated	334	70.9	321	69.0	60	70.6	50	56.8
Treatment completed	168	35.7	175	37.6	38	44.7	30	34.1
Planned treatment	87	18.5	88	18.9	27	31.8	24	27.3
**Recurrence** (yes)	115	20.9	88	18.9	27	31.8	26	29.5
**Other diseases** (yes)	263	55.8	280	60.2	46	54.1	41	46.6
**Secondhand smoke**								
Yes	88	18.7	95	20.4	37	43.5	54	61.4
	M (SD)		M (SD)		M (SD)		M (SD)	
**Health status (last week)**^a,b^	4.50 (1.55)		4.37 (1.51)		3.82 (1.53)		4.42 (1.62)	
**Quality of life (last week)**^b^	4.60 (1.59)		4.49 (1.54)		3.96 (1.58)		4.60 (1.69)	

^a^EORTC Item 29

^b^EORTC Item 30; 1 = very bad; 7 = excellent

^c^Self-assessment allowed classification into multiple responses, e.g., had surgery, planned to undergo chemotherapy

RQ1: What is the proportion of cancer patients who smoke, and how can their smoking patterns be characterized?

In our sample the prevalence of CS was 7.9% (*n*=88 CS out of *n*=1.109 total participants) with a confidence interval of 6.3% - 9.7%. The proportion of patients who continued to smoke after diagnosis was 50.9% (*n*=88 CS of *n*=173 combined CS and EX-after). The vast majority of former smokers (Ex-after) quit within the first year after diagnosis.

On average, current smokers have smoked for 39.65 (SD=11.47) years, ranging from 10 to 58 years (see Table 3). None of the smokers had started smoking after their current cancer was diagnosed. The number of cigarettes smoked per day varies widely, with a mean of M=10.85 (SD=9.27). This results in a mean of M=21.51 pack-years. Of all smokers, 15 participants reported smoking only alternatives to cigarettes, such as cigars, cigarillos, and pipes. Furthermore, 31.8% (*n*=28) of CS reported to have temporarily quit smoking, all of them before diagnosis. Their smoking abstinence lasted approximately two years (median). For the analysis of the Fagerström test for nicotine dependence due to cigarette smoking, ten patients were excluded because they had more than 30% missing values. Of the remaining 63 current cigarette smokers, 33.3% have low, 41.3% medium, 25.4% high or very high dependence.

**Table 3:** Smoking patterns of CS

	**CS (*N* = 88)**	
	**N**	**%**
**Smoking product**		
Cigarette smoking	73	83
Smoking only alternative products	15	17
	**M**	**SD**
**Number of cigarettes per day** (*n* = 73)	10.85	9.27
**Number of e-cigarettes per day** (*n* = 6)	14.00	3.74
**Number of cigarillos/cigars/pipes per day** (*n* = 14)	4.48	4.97
**Smoking years**	39.65	11.47
	**N**	**%**
**Nicotine dependence due to cigarettes** (*n* = 63)^a^		
Low	21	33.3
Medium strong	26	41.3
High/very high	16	25.4
**Motivational Stage of Change** (*N* = 88)		
Lack of intention	16	18.2
Intention	52	59.1
Action	20	22.7

^a^Patients excluded due to more than 30% missing values

RQ 2: What sociodemographic, medical, and psychological factors are associated with current smoking status after a cancer diagnosis?

Educational level was dichotomized prior to analysis (highest German school degree vs. lower degrees). We further reduced the cancer type category by clustering it according to its association with tobacco, with the categories” tobacco-associated” or “not tobacco-associated”. Three patients were excluded from the analysis due to more than 30% missing values in any of the predictor variables. Multicollinearity analyses in this and the following two regression models yielded a VIF≤ 1.51, indicating that there were no multicollinearity concerns.

The results of the logistic regression analysis for predicting smoking cessation after cancer diagnosis are shown in Table 4. A diagnosis of a tobacco-related cancer type increases the odds of quitting smoking (OR=2.781, 95%CI=1.241;6.230). No other associations were found.

**Table 4:** Prediction of smoking cessation after a diagnosis of cancer (multivariate logistic regression)

	**EX-after**	
	**OR**	**[CI 95%]**
**Variables**		
Gender (male:72%)	0.467	[0.207;1.057]
Age	0.975	[0.946;1.005]
Education (at least high school diploma: 48%)	1.198	[0.591;2.430]
Relationship (23%: no relationship)	1.029	[0.444;2.381]
Having children (yes : 71%)	1.128	[0.524;2.428]
Tobacco associated cancer type (yes: 25%)	2.781*	[1.241;6.230]
Alcohol consumption	0.921	[0.789;1.076]
Positive support (SSUK)	0.963	[0.856;1.083]
Negative interactions (SSUK)	1.053	[0.955;1.161]

^*^p < .05; *n* = 170; Nagelkerke *R*^2^ = .166, reference category: CS

RQ 3: What sociodemographic, medical, and psychological factors are associated with the level of nicotine dependence in current smoking cancer patients?

Due to the small sample size and unequal group sizes the criteria levels “severe” and “very severe dependence” were combined into one level “severe to very severe dependence”. *N*=15 patients were excluded because they reported smoking only nicotine-containing cigarette alternatives (see RQ1). Eleven patients were excluded from the analyses due to more than 30% missing values in any of the predictor variables.

Results are shown in Table 5: A diagnosis of tobacco-related cancer increased the odds of medium dependence compared to low dependence (OR=8.903, CI=1.064;74.464). Having more positive support in stressful situations (SSUK) predicted severe to very severe dependence compared to low dependence (OR=1.415, CI=1.065;1.879). No other significant associations were found.

**Table 5:** Prediction of nicotine dependence (multinomial logistic regression) among current cigarette smokers (CS subsample)

	**Fagerström**			
	**Medium dependence**		**Severe – very severe dependence**	
**Variables**	**Odds ratios**	**[CI 95%]**	**Odds ratios**	**[CI 95%]**
Gender (male: 81%)	0.264	[0.024; 2.884]	0.456	[0.031; 6.502]
Age	1.043	[0.978; 1.112]	1.022	[0.948; 1.101]
Education (at least high school diploma: 56%*)*	1.556	[0.334; 7.236]	4.961	[0.676; 36.36]
Relationship (no:19%)	2.070	[0.186; 23.04]	15.536	[0.941; 256.48]
Having children (yes : 71%)	1.349	[0.251; 7.251]	1.087	[0.149; 7.876]
Tobacco associated cancer type (yes: 21%)	8.903*	[1.064; 74.464]	6.121	[0.513; 73.034]
Alcohol consumption	0.898	[0.663; 1.214]	0.676	[0.449; 1.016]
Positive support (SSUK)	1.176	[0.940; 1.470]	1.415*	[1.065; 1.879]
Negative interactions (SSUK)	1.044	[0.822; 1.326]	1.181	[0.853; 1.633]

^*^p < .05; *n* = 62; Nagelkerke *R*^2^ = .43; reference category: low dependence

RQ 4: What is the proportion of cancer patients who continue to smoke in each motivational stage of the adapted version of the TTM (lack of intention, intention formation and action), and what sociodemographic, medical, and psychological factors are associated with each stage??

Of all cancer patients who smoked 18.2% (*n*=16) have no intention to quit, 59.1% (*n*=52) have an intention to quit, and 22.7% (*n*=20) are already taking steps to reduce or stop smoking (see Table 6). *N* = 12 had to be excluded from the regression analysis due to more than 30% missing values in any of the predictor variables.

**Table 6:** Associations with motivational change (multinomial regression) of CS

	**Stages of change**			
	**Lack of intention**		**Intention formation**	
**Variables**	**Odds ratios**	**[CI 95%]**	**Odds ratios**	**[CI 95%]**
Gender (male:83%)	2.853	[0.275; 29.564]	2.600	[0.417; 16.197]
Age	0.972	[0.906; 1.042]	1.012	[0.955; 1.070]
RELATIONSHIP (no:22%)	4.029	[0.372; 43.545]	3.139	[0.451; 21.844]
Having children (yes:71%)	1.447	[0.190; 10.999]	0.674	[0.157; 2.894]
Tobacco associated cancer type (yes:16%)	2.058	[0.134; 31.488]	5.237	[0.637; 42.988]
Alcohol consumption	0.885	[0.622; 1.259]	0.837	[0.634; 1.104]
Knowledge on the effects of continued smoking after cancer	0.850	[0.706; 1.023]	0.959	[0.832; 1.104]

*n* = 76; Nagelkerke *R*^2^ = .194; reference category: action

No significant association was found between the predictor variables analyzed and the stage of motivational change (Table 6).

Sensitivity analyses (complete cases without imputation of missing values) of all inferential statistics (RQ 3 and 4) showed similar results.

RQ 5: What is the perceived need for a specific smoking cessation program for cancer patients and how should this program be designed?

Former smokers (EX-before; EX-after) answered four questions and current smokers (CS) answered five questions about their opinion of a smoking cessation program for cancer patients (see Table 7).

**Table 7:** Patients’ opinions of a smoking cessation program (by subgroup)

	**Never smokers (NS) (*N* = 471)**	**EX-before (*N* = 465)**	**EX-after (*N* = 85)**	**CS (*N* = 88)**
	**M (SD)**	**M (SD)**	**M (SD)**	**M (SD)**
**Opinion on a smoking cessation program for cancer patients**				
1. Education/information is important	n.a	4.39 (0.94)	4.25 (0.98)	4.00 (0.89)
2. It makes sense to offer a special smoking cessation program for cancer patients	n.a	4.39 (0.92)	4.26 (0.90)	4.01 (0.80)
3. Offer smoking cessation specific to patients with similar types of cancer	n.a	2.70 (1.40)	2.74 (1.35)	2.92 (1.13)
4. Smoking cessation program at treatment site	n.a	3.94 (1.05)	3.90 (0.92)	3.61 (0.91)
5. Willingness to participate in a smoking cessation program (only CS)	n.a	n.a	n.a	3.12 (1.10)

Response options are: 1 = not true at all, 2 = rather not true, 3 = neutral, 4 = is rather true, 5 = is very true

Education and information about different ways to quit, the availability of such a program specifically for cancer patients and the availability of such a program at the site of treatment are considered as rather important. The availability of a specific program for similar tumor groups was considered indifferent. The proposed willingness of smokers to participate in a smoking cessation program was rated as neutral.

CS answered five more specific questions about the design of a smoking cessation program. Missing values were common for questions about the maximum amount of money they would be willing to spend on such an intervention, as well as the preferred time of day, frequency, and setting. Over half of the cancer patients who smoked were willing to spend up to €100 for the intervention. Most patients (37.0%) indicated that they would prefer or would only attend an evening program, followed by 27.4% who would prefer a morning program or would not mind either time (Table 8).

**Table 8:** Suitable design of a smoking cessation program

	**CS**	
	**N**	**% (of cases)**
**The best time for me to attend a smoking cessation program is …** (*n* = 73 cases/*n* = 83 responses)		
Morning	20	27.4
Afternoon	16	21.9
Evening	27	37.0
Does not matter	20	27.4
**How often should the program take place?** (*n* = 57 cases/*n* = 60 responses)		
1-3x	17	29.8
3-5x	19	33.3
> 5	7	12.3
Does not matter	17	29.8
**What setting should the program run in?** (*n* = 67 cases/*n* = 96 responses)		
Group	36	53.7
Online/app	24	35.8
Single	25	37.3
Do not care	11	16.4
**Willingness to pay for a cessation program for cancer patients?** (*n* = 55)		
Up to 50 Euro	18	32.7
Up to 75 Euro	2	3.6
Up to 100 Euro	16	29.1
Up to 125 Euro	2	3.6
Up to 150 Euro	6	10.9
Up to 175 Euro	2	3.6
Up to 200 Euro	4	7.3
More than 200 Euro	5	9.1

When asked how often a program should take place, one-third of patients would prefer meetings up to three times per week, and another third would prefer up to five times per week.

When asked about their preferred setting for a smoking cessation program, patients were given a choice between group, online/app-based or one-on-one sessions.

Regarding the setting, 53.7% of patients would participate in group sessions, followed by one-on-one sessions (37.3%) and online/app-based sessions (35.8%).

## Discussion

In this study, we examined smoking patterns among cancer patients and their sociodemographic, medical, and psychosocial associations in a large metropolitan region in Germany. The overall aim was to understand potential cornerstones for the implementation of an effective and sustainable smoking cessation program for cancer patients that considers specific needs of this group. In our sample, half of the smoking cancer patient population quit smoking after being diagnosed with cancer, while the other half continued to smoke. The vast majority of former smokers quit within the first year after diagnosis, while some patients did not quit until many years after their cancer diagnosis. Both of these findings are consistent with previous literature: Studies show that up to 60 percent of cancer patients continue to smoke after cancer diagnosis and that it takes up to 7.5 years to successfully quit smoking [13, 16, 18]. The results of our study show that there is an urgent need for smoking cessation support in the German cancer population, as indeed a large number of cancer patients who smoke could benefit from it.

Also, the duration of smoking among cancer patients in this cohort was almost 40 years on average and surprisingly no patient was assessed with less than ten years of smoking. A study by Kim et al. 2014 showed that the duration of smoking was positively associated with continued smoking after a cancer diagnosis. Since in our population many of the smoking cancer patients had already smoked for a long time, this aspect should be given special attention when developing a smoking cessation program. People with long smoking histories have often started smoking at a young age and we already know for the general population that a younger start, before the age of 20, increases the likelihood of nicotine dependence compared with a later start [34].

When designing a targeted smoking cessation program for cancer patients, it is also important to consider individuals with different smoking levels and different smoking products. On average, patients in the study cohort smoked approximately 11 cigarettes per day, with some smoking as little as one cigarette per week and others smoking up to 58 cigarettes per day. In addition, 17.0% of the smoking population smoked nicotine-containing cigarette alternatives (e-cigarettes, cigars). Especially in view of the increased use of e-cigarettes by cancer patients in the coming years and more data and medically solid information and recommendations on the use of e-cigarettes by cancer patients, this should be taken into serious consideration in future smoking cessation programs. Overall, in order to inform and involve all cancer patients, an intervention should therefore provide information about the various tobacco products and not just focus on cigarettes.

While 30.0% of the smokers in our population reported having taken a break from smoking, and this break lasted approximately 2 years, it would be interesting to understand what caused this break, and how professionals could recognize and use this as a window of opportunity to help smokers quit successfully.

Interestingly, one third of CS had low cigarette dependence as measured with the FTCD but continued to smoke after being diagnosed with cancer. Typically, cancer patients with high dependence are less likely to quit smoking than smokers with low dependence [35]. It could therefore be speculated that there is still a lack of motivation in this cohort or that there has not been sufficient education about the consequences of continuing to smoke as well as motivational interviewing to increase the level of desire to quit.

Our study revealed two significant findings. First, smokers with a tobacco-associated cancer diagnosis were more likely to be nicotine dependent than smokers with a cancer diagnosis not typically associated with smoking. Although the data are only cross-sectional, it is very plausible to assume that the inability to quit smoking increased the risk of developing a tobacco-related tumor. Second, these patients with a tobacco-associated cancer diagnosis in our study were also more likely to quit smoking after diagnosis than smoking cancer patients without a tobacco-associated tumor. One explanation could be that patients with a tobacco-related tumor are more likely to be aware, or better informed by oncology staff, that smoking has a detrimental effect on the development and treatment of their cancer, so they are more likely to be able to stop smoking after diagnosis. This would have several implications for the development of a smoking cessation intervention for cancer patients. For those with non-tobacco related tumors, the intervention should focus on education, motivation to quit smoking, and the possible use of a smoking cessation program.

In our study higher cigarette dependence was associated with more positive social interactions, such as social support or positive interactions during cancer treatment or follow-up. The importance of social support for cancer patients, especially for smoking cessation, is essential. Other studies have shown that cancer survivors who experienced higher levels of social support were less likely to become smokers [19] and cancer survivors who rated their support system as rather low were more likely to continue smoking after diagnosis [36]. As our study shows conflicting results, the question arises as to whether support can also have a negative effect, i.e. whether it may even make someone more likely to continue smoking after diagnosis. One hypothesis might be that cancer smokers feel unconditionally supported even if they continue to smoke and are clearly harming themselves by doing so. They may also have many positive interactions with other smokers in their supportive social environment. It is possible that cancer smokers would benefit from positive support related to coping with the cancer diagnosis, but also from receiving a clear message to quit smoking from their supportive environment. Cancer smokers who want to quit should also be encouraged to stop associating with people who encourage smoking because they smoke. Regarding the stages of change (TTM) of motivation to quit smoking, more than half of the smoking patients indicated that they were in the “intention formation” phase. About another quarter of patients was already taking action to quit smoking, while the remaining patients showed a lack of intention to quit smoking. Not surprisingly, research on the stage model suggests that people who are taking action are more likely to be abstinent 6-12 months after a brief smoking cessation intervention [37, 38]. Accordingly, the goal should be to provide specific interventions depending on the motivational phase so that everyone ends up taking action. To this end, the motivational phase of smokers should be identified in routine clinical practice. In our study we were not able to find an association between knowing more about the harmful consequences of continued smoking and being in a specific state of the TTM.

However, to our knowledge, this is the first study using a standardized questionnaire (KSC-8) to assess knowledge of the impact of smoking on cancer treatment in cancer patients. Even if no effects have been found in this study, it is still likely, that increased smoking knowledge can increase motivation to quit, and therefore this potential should be exploited. Education should therefore be provided directly by the oncology staff caring for the patient. To date, there has been too little discussion in oncology clinics about smoking and smoking education for cancer patients. In previous studies of cancer patient education, only about half of cancer patients reported receiving any information about the consequences of continuing to smoke after their cancer diagnosis [13]. In a survey of oncology professionals, although almost all reported that tobacco cessation was an important part of cancer care, only few of them routinely provided smoking cessation support [39].

The results of this study provide a first insight into the smoking patterns of German cancer patients and underline the need for patient education and smoking cessation services in German oncological cancer centers. The identified associations between smoking behavior and sociodemographic, psychological, and medical factors need to be taken into account in the development of these services in order to tailor them to the needs of this target group.

### Limitations

Some potential limitations need to be discussed. First, we have refrained from using a minimum abstinence period for former smokers to be classified as “former smokers”. In practice, this allows patients to subjectively decide whether they still consider themselves as smokers or former smokers. Our rationale for this decision can be summarized as follows: There is still no clear definition of the length of time after which a patient achieves long-term abstinence without relapse. Segan et al [40] analyzed relapse in smokers during a six-months period after quitting. They found that the reported temptation to smoke decreased over time and already stabilized after about one month of abstinence, while others recommend at least six months of abstinence [41]. However, several longitudinal studies even suggest that a substantial number of quitters relapse years after quitting [42–45]. Smoking relapse during the first year after cessation is particularly common in cancer patients [43, 45]. Therefore, there is a need to further investigate different durations of abstinence that are associated with a high probability of sustained abstinence specifically in cancer patients. Smoking cessation programs need to be continued in the follow-up of cancer patients in the years after the end of treatment and may even be valuable for smoking cessation at any time.

Second, the study was conducted using a cross-sectional design. Therefore, causal inferences are limited [46]. Nevertheless, our cross-sectional design included patients at different stages of disease and treatment in order to capture different motivational stages. In addition, a cross-sectional design has several advantages over a longitudinal design: It is easier to recruit a sufficient number of patients, which limits the burden on participating patients, and ensures anonymity.

Third, we do not expect smokers to classify themselves as smokers after a very short period of abstinence. Most smokers have experience with quit attempts and relapse [42, 44, 45]. This was also confirmed in interviews with patients for content validation of the KSC-8. All patients immediately identified themselves as former smokers or current smokers as mentioned above.

Although it is not certain, that the former smokers identified by our classification will remain permanently abstinent, there is also no defined period of time that guarantees long-term abstinence and prevention of relapse in former smokers.

Another limitation is the focus on cigarettes in this study. Although data on the use of other smoking products such as cigars, cigarillos and pipes are examined, they are presented only descriptively. For the sake of simplicity, we did not focus on the potentially different nicotine concentrations in both products (e-cigarettes and cigarettes) and brands [47, 48].

Furthermore, critical items measuring smoking dependence and motivation to quit smoking (FTCD, FÄR) were found to have missing values of about 12%. It can be assumed that smoking and motivation to quit smoking are associated with shame, especially in cancer patients. Despite anonymity, they may have felt uncomfortable, not wanted to be confronted with their own negative behavior or feared stigmatization.

With respect to the cancer population studied, older male patients were overrepresented in the overall sample, especially in the smoking subsample and urogenital cancers were also represented at an absolute higher rate explained by the fact, that patients were also recruited from a specific prostate cancer center, which is was part of the network of the comprehensive cancer center. Overall, this can be considered to be the major limitation of this study. Therefore, the question arises to what extent it is possible to draw conclusions about the general cancer population of smokers from the sample studied. The recruited prostate cancer patients are exclusively men who are mainly treated by one surgical removal of the tumor and are therefore only restricted in their mobility for a few days. These patients might therefore be in a much better position to participate in, travel to, and physically endure the progress of a smoking cessation intervention. Therefore, factors that appear to be important in this population may not apply to other cancer patients who are already much more limited by disease, metastasis, and type of treatment. Nevertheless, these are important initial findings on the smoking behavior of German cancer patients that can be used to develop interventions that benefit smoking cancer patients in quitting smoking. Further studies should focus more on smoking cancer patients who are less mobile and may need interventions directly located at their treatment site.

## Conclusion

In summary, this study shows that about half of cancer survivors continue to smoke after a cancer diagnosis, although only a small proportion are by definition highly dependent on cigarettes. Cancer patients smoke a variety of different smoking products in large quantities and have a long smoking history. Educating smokers about all types of harmful products must be an essential part of a smoking cessation intervention. The window of opportunity after a cancer diagnosis must be recognized by clinicians and used to motivate patients to quit smoking in an intervention. In particular, patients without a tumor-associated cancer diagnosis need to be motivated and educated about the consequences of smoking. However, in a smoking cessation intervention, patients with a tobacco-associated tumor diagnosis must also be supported to quit, as they may show signs of higher nicotine dependence. Although more than half of cancer patients are already in the intention formation phase, a smoking cessation program must also focus on engaging all smokers in different motivational phases.

These findings may provide important considerations for developing a tailored smoking cessation program to help cancer patients quit smoking.

### Supplementary Information

Supplementary Material 1.

Supplementary Material 2.

## Data Availability

All relevant data are included in this publication. Detailed information will be provided upon reasonable request, e.g., for systematic reviews or meta-analyses. Please contact f.bokemeyer@uke.de for more details.

## Abbreviations

AUDIT: Alcohol use disorder identification test
AUDIT-C: Alcohol use disorder identification test Consume
BGS98: German health survey
CI: Confidence interval
CS: Current smoker
Ex-after: Ex-smokers, who stopped after cancer diagnosis
Ex-before: Ex-smokers, who stopped before cancer diagnosis
EORTC QLQ C30: European organization for research and treatment of cancer quality of life questionnaire
FÄR: Questionnaire for measuring the intention to quit smoking
FS: Former smoker (someone who has given up smoking)
FTCD: Fagerström Test for Cigarette Dependence
GNC: German National Cohort
HRQOL: Health related quality of Life
KSC-8: Knowledge of smoking after cancer
LPEK: Local psychological Ethic committee at the center of psychosocial medicine Hamburg, Germany
M: Mean
NS: Never smokers
OD: Odds ratio
OSCC: Opinion on a smoking cessation program for cancer patients
OSF: Open Science Framework
QOL: Quality of Life
RQ: Research question
SD: Standard deviation
SPSS: Statistical Package for the Social Sciences
SSUK-8: 8-Item abbreviated version of the social support with illness scales in cancer patients
TTM: Transtheoretical Model
VIF: Variance inflation factor
