# Supplementary material for: Smoking patterns and the intention to quit in German cancer patients: a cross-sectional study
Source: BMC Cancer. 2024 Jun 6;24:693. doi: 10.1186/s12885-024-12380-w (PMC11155111; doi:10.1186/s12885-024-12380-w)
Supplement: Supplementary file 1 — Supplementary Material 1. [file 12885_2024_12380_MOESM1_ESM.docx]

# **KSC-8: Knowledge of smoking after cancer.**

| **Connection between cancer and smoking** | | | | | | |
| --- | --- | --- | --- | --- | --- | --- |
| The following statements address the connection between cancer and smoking. We would like to learn how we can better inform and educate our patients. If you are unsure of an answer, please check the answer choice that best fits your assessment. Please do not omit any question. | | | | | | |
|  | | **strongly disagree** | **disagree** | **undecided** | **agree** | **strongly agree** |
| 1. | It makes little sense for cancer patients to stop smoking because they already have cancer. | ⬜ | ⬜ | ⬜ | ⬜ | ⬜ |
| 2. | Smoking can adversely affect various cancer therapies (e.g., radiation therapy). | ⬜ | ⬜ | ⬜ | ⬜ | ⬜ |
| 3. | The quality of life of lung cancer patients who stop smoking after cancer diagnosis is higher in the long term compared to lung cancer patients who continue to smoke. | ⬜ | ⬜ | ⬜ | ⬜ | ⬜ |
| 4. | For several cancers, surgery risks are increased for patients who smoke. | ⬜ | ⬜ | ⬜ | ⬜ | ⬜ |
| 5. | Continued smoking leads to weight gain in cancer patients. | ⬜ | ⬜ | ⬜ | ⬜ | ⬜ |
| 6. | The effectiveness of cancer chemotherapy may be increased in patients who continue to smoke. | ⬜ | ⬜ | ⬜ | ⬜ | ⬜ |
| 7. | The course of cancer can be much more aggressive in smokers. | ⬜ | ⬜ | ⬜ | ⬜ | ⬜ |
| 8. | For some cancers, continuing to smoke can increase the risk of developing a second tumor. | ⬜ | ⬜ | ⬜ | ⬜ | ⬜ |

Note: After submitting the completed questionnaire, all patients receive a clarification of the correct and incorrect information.
