# Supplementary material for: Smoking patterns and the intention to quit in German cancer patients: a cross-sectional study
Source: BMC Cancer. 2024 Jun 6;24:693. doi: 10.1186/s12885-024-12380-w (PMC11155111; doi:10.1186/s12885-024-12380-w)
Supplement: Supplementary file 2 — Supplementary Material 2. [file 12885_2024_12380_MOESM2_ESM.docx]

### OSCC: Opinion on a smoking cessation program for cancer patients

**For former smokers:**

| In the following, we are interested in your opinion about smoking cessation. | | | | | | |
| --- | --- | --- | --- | --- | --- | --- |
|  | | **strongly disagree** | **disagree** | **undecided** | **agree** | **strongly agree** |
|  |  | **1** | **2** | **3** | **4** | **5** |
| 1. | I think education/information about different ways to quit smoking in cancer patients is important. | ⬜ | ⬜ | ⬜ | ⬜ | ⬜ |
| 2. | I think offering a special smoking cessation program for cancer patients makes sense. | ⬜ | ⬜ | ⬜ | ⬜ | ⬜ |
| 3. | I think offering smoking cessation program that only includes patients with comparable tumor types makes sense (e.g., breast cancer, lung cancer, etc.) | ⬜ | ⬜ | ⬜ | ⬜ | ⬜ |
| 4. | I find it useful to being offered a smoking cessation program directly at the point of treatment (e.g., at the hospital or in my doctor's office). | ⬜ | ⬜ | ⬜ | ⬜ | ⬜ |

**For current smokers:**

| In the following, we are interested in your opinion about smoking cessation. | | | | | | | | | | | |
| --- | --- | --- | --- | --- | --- | --- | --- | --- | --- | --- | --- |
|  |  | **strongly disagree** | **disagree** | | | | **undecided** | **agree** | | | **strongly agree** |
| 1. | I think education/information about different ways to quit smoking in cancer patients is important. | ⬜ | ⬜ | | | | ⬜ | ⬜ | | | ⬜ |
| 2. | I think offering a special smoking cessation program for cancer patients makes sense. | ⬜ | ⬜ | | | | ⬜ | ⬜ | | | ⬜ |
| 3. | I think offering smoking cessation program that only includes patients with comparable tumor types makes sense (e.g., breast cancer, lung cancer, etc.) | ⬜ | ⬜ | | | | ⬜ | ⬜ | | | ⬜ |
| 4. | I find it useful to being offered a smoking cessation program directly at the point of treatment (e.g., at the hospital or in my doctor's office). | ⬜ | ⬜ | | | | ⬜ | ⬜ | | | ⬜ |
| 5. | I could see myself participating in a smoking cessation program for cancer patients. | ⬜ | ⬜ | | | | ⬜ | ⬜ | | | ⬜ |
| 6. | A smoking cessation program must be easy to integrate into everyday life: | **yes** | | | **no** | | | | **I do not care** | | |
|  | Timewise it suits me best ... |  |  |  |  |  |  |  |  |  |  |
|  | In the morning | ⬜ | | | ⬜ | | | | ⬜ | | |
|  | in the afternoon | ⬜ | | | ⬜ | | | | ⬜ | | |
|  | in the evening | ⬜ | | | ⬜ | | | | ⬜ | | |
|  | I can/would like to attend at most ... appointments |  | | | | | | | | | |
|  | 1-3 appointments | ⬜ | | | ⬜ | | | | ⬜ | | |
|  | 3-5 appointments | ⬜ | | | ⬜ | | | | ⬜ | | |
|  | more than 5 appointments | ⬜ | | | ⬜ | | | | ⬜ | | |
| 7. | I can think of the following setting for smoking cessation for me: |  | | | | | | | | | |
|  | in a group | ⬜ | | | | ⬜ | | | | ⬜ | |
|  | online/ in an app | ⬜ | | | | ⬜ | | | | ⬜ | |
|  | solo setting | ⬜ | | | | ⬜ | | | | ⬜ | |
| 8. | I would be willing to pay the following fee for participating in a smoking cessation specifically for cancer patients: | ⬜ | | Up to 50 euro | | | | | | | |
|  |  | ⬜ | | Up to 75 euro | | | | | | | |
|  |  | ⬜ | | Up to 100 euro | | | | | | | |
|  |  | ⬜ | | Up to 125 euro | | | | | | | |
|  |  | ⬜ | | Up to 150 euro | | | | | | | |
|  |  | ⬜ | | Up to 175 euro | | | | | | | |
|  |  | ⬜ | | Up to 200 euro | | | | | | | |
|  |  | ⬜ | | More than 200 euro | | | | | | | |
